# Diffusion of Nanorods with Various Lengths and Rigidities in Cross-Linked Networks

**DOI:** 10.3390/polym18010003

**Published:** 2025-12-19

**Authors:** Bin Li, Pingcuozhuoga

**Affiliations:** 1 School of Chemical Engineering and Technology, Sun Yat-sen University, Zhuhai 519082, China; 2 State Grid Xizang Electric Power Research Institute, Lhasa 850000, China

**Keywords:** coarse-grained simulation, anomalous diffusion, anisotropic diffusion

## Abstract

We investigated diffusion of thin and thick nanorods with varying lengths and rigidities in cross-linked polymer networks using coarse-grained molecular dynamics (CGMD) simulations. Our results show that the translational diffusion of nanorods slows down with power scaling laws as their length increases, exhibiting a non-monotonic dependence on rigidity of thin nanorods, and decreases with the rigidity of thick nanorods. The sub-diffusion of nanorods is observed at short time scales, which becomes more pronounced for rigid nanorods. The nanorods show anisotropic diffusion behavior with favoring motion along their major axes in cross-linked networks, and the anisotropy enhances by increasing either rigidity or length of nanorods, especially for thick nanorods. The sub-diffusion behavior of nanorods is primarily due to the strong heterogeneity of motions perpendicular to major axes of nanorods, and the time scales of this heterogeneous diffusion increase with the length and rigidity of nanorods. In rotational dynamics, nanorods with higher rigidity rotate more slowly, and the effect is more evident in longer nanorods. The rotational diffusion coefficient follows a power scaling law with the rigidity of nanorods, when the effective length of a nanorod exceeds the mesh size of cross-linked network. The rotations of nanorods also display heterogeneous dynamics, in which the time scale of heterogeneous rotation increase with rigidity, and such heterogeneity is more pronounced in softer nanorods. Overall, our work elucidates the microscopic mechanisms governing both translational and rotational diffusion of nanorods in cross-linked networks.

## 1. Introduction

In 1827, Brown observed that particles exhibit continuous and irregular motion in water. Subsequently, Einstein and Smoluchowski developed statistical explanations for the diffusion of microscopic particles, which formulated the diffusion theory for microscopic particles in a uniform solvent. According to the Stokes–Einstein relation, the diffusion of microscopic particles, such as colloids, in a dispersion medium is inversely proportional to the particle radius and the viscosity of the dispersion medium. These diffusion theories laid the foundation for subsequent research by numerous scientists on particle diffusion in both simple solutions and complex systems.

Diffusion processes have been recognized as playing a vital role in soft matter and biological systems [1,2,3], such as the targeted therapy provided by the delivery of nano-carriers in organisms [4]. According to the aforementioned Stokes–Einstein relation, the diffusivity of microscopic particles in a dispersion medium is closely related to their size. However, in complex environments such as mucus and cytoskeleton, which contain cross-linked gel networks with protein fibers of certain persistence lengths, such a semi-flexible network structure can hinder the diffusion of microscopic particles, leading to the anomalous diffusion behavior. Moreover, the diffusion mechanism of microscopic particles is influenced by the relationship between their size and the mesh size of the network [5,6]. Over the past decades, numerous experimental and theoretical studies have explored the diffusion of nanoparticles in gel-like networks [7,8]. For instance, Hanes and co-workers demonstrated that coating nanoparticles with polyethylene glycol (PEG) enhances their diffusion through mucus [9,10,11]. Yan and co-workers investigated the diffusion behavior of nanoparticles in the cell membranes and mucus cross-linked networks and discovered that diffusion of nanoparticles exhibits multiple mechanisms, including Brownian motion, Lévy flight, directional motion, and so on, depending on the properties of nanoparticles and their local environment [12,13,14,15,16,17].

According to the previous studies by Yu et.al., the diffusion behaviors of nanoparticles are significantly influenced by their shapes and rigidities [18,19,20,21]. For instance, rod-shaped nanoparticles exhibit faster dynamics in mucus cross-linked network than spherical nanoparticles of identical effective size [18,22,23]. Moreover, the introducing of shape anisotropy in rod-shaped nanoparticles results in more complex diffusion mechanisms. First, the diffusion of nanorods also exhibits potential anisotropic effect due to their high aspect ratio [24,25,26,27,28]. Second, the anisotropic shape inevitably slows down rotational dynamics, which is attributed to the steric hindrance in complex environments, creating an additional free energy barrier for the rotation of nanorods [29]. Therefore, both translational and rotational diffusion of nanorods with certain aspect ratios tend to exhibit heterogeneous dynamics in cross-linked networks [26].

The rigidity of nanorods also provides another influence factor for their diffusion. Compared to fully rigid ones, soft nanorods exhibit a shorter effective length, yet appear to possess a larger effective cross-sectional area. Consequently, the rigidity of nanorods influences both the translational and rotational diffusion within cross-linked networks. Moreover, as the relationship between mesh size of cross-linked network and the size of nanoparticles is critical for the diffusion of nanoparticles in complex media [6,29], the effective length and cross-sectional area of nanorods governed by their rigidities are expected to affect their diffusion in such environments. In addition, the diameter of nanorods provides another influence factor on their anisotropic and rotational dynamics. Therefore, to elucidate the underlying mechanisms governing the diffusion of nanorods with varying lengths and rigidities in cross-linked networks, we conducted extensive molecular dynamics simulation to investigate both the translational and rotational diffusion behavior of nanorods. By analyzing the microscopic diffusion mechanism of nanorods with different properties in cross-linked networks, this work aims to provide feasible strategies for designing functional nanoparticles for drug delivery and other applications.

## 2. Model and Method

**Figure 1:**
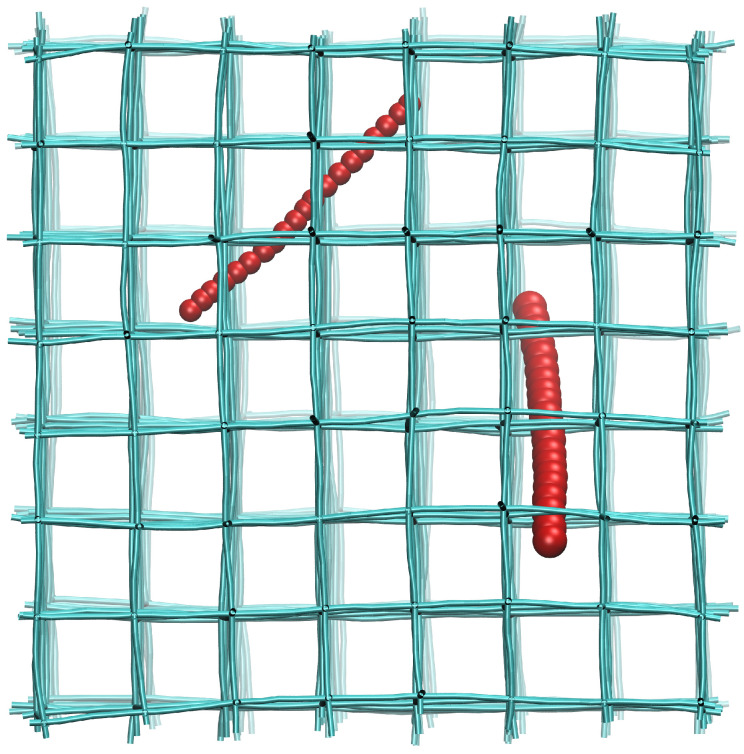
Schematic illustration of the cross-linked network (cyan) and nanorods (red). The left and right nanorods represent thin and thick nanorods, respectively.

We employed coarse-grained molecular dynamics (CGMD) simulations to investigate the diffusion of nanorods in cross-linked networks. Both the network and nanorod were constructed using bead-spring CG model. The cross-linked network, built as a cubic lattice with a mesh size of $a_{x}=6\sigma$ (where σ is the length unit), consists of 9016 CG beads. The detailed methodology for constructing the network is described in our previous work [30]. Each nanorod was modeled as a linear chain of CG beads, with the numbers of CG beads *N* set to 10, 15, 20 and 25, to represent the various lengths of nanorods. The methodology for building nanorods with different flexibility has been adopted in some previous work [24,31]. A schematic diagram of our simulation system is shown in Figure 1.

The pairwise interactions between CG beads are represented using Lennard-Jones (LJ) potential,

(1) $$U_{\mathrm{LJ}}=4\varepsilon_{ij}\left[{\left(\frac{\sigma_{ij}}{r}\right)}^{12}-{\left(\frac{\sigma_{ij}}{r}\right)}^{6}\right]$$

where $\varepsilon_{ij}$ is the interaction parameter between CG beads *i* and *j*, and $\sigma_{ij}$ is the distance where the potential equals to zero. Here we set $\varepsilon =0.1\;k_{B}T$ for the LJ interactions between all the pairs of CG beads, where $k_{B}$ is the Boltzmann constant, and *T* is the system temperature fixed at 1.0. The value of $\varepsilon =0.1\;k_{B}T$ reflects a essentially relative repulsion, according to its relation with second virial coefficient [32]. This value was adopted in some previous studies [19,20,33,34], and it is close to the interaction between nanoparticles coated by hydrophilic polymers, like PEG, and other soft materials like ionic liquids, mucin, etc. [19,35,36]. We set $\sigma_{nn}=1\sigma$ for the LJ potential between CG beads in nanorods (denoted by subscript *n*) and $\sigma_{cc}=1\sigma$ for interactions between cross-linked networks beads, with σ being the length unit in our simulations. We set two distinct $\sigma_{cn}$ values for the interaction between nanorod beads and network beads, and $\sigma_{cn}$ was set to 2σ for thin nanorods, yielding an effective nanorod diameter of 3σ, according to Lorentz–Berthelot mixing rule. To represent thick nanorods, we doubled the effective cross-sectional area, corresponding to an effective diameter of $3\sqrt{2}\sigma$. This leads to a value of $\sigma_{cn}\approx 2.62\sigma$ for the Lennard-Jones potential between thick nanorods and network beads. The use of larger $\sigma_{cn}$ values helps to capture the rod-like geometry of the coarse-grained chains, for representing nanorods with different effective lengths. The cutoff radius is 2.5$\sigma_{ij}$, at which the LJ potential is truncated and shifted to zero.

Two adjacent beads in the cross-linked network and nanorods are covalently bonded via a harmonic bond potential,

(2) $$U_{b}=k_{b}{\left(r-r_{0}\right)}^{2}$$

where $k_{b}$ is the bond spring constant, and $r_{0}$ is the equilibrium bond length. Here, we set $k_{b}=25\;k_{B}T/\sigma^{2}$ and $r_{0}=1\sigma$. The non-bonded LJ interactions between bonded beads are excluded. The rigidity of the network and nanorods is controlled by a three-body angle potential,

(3) $$U_{a}=k_{a}{\left(\theta -\theta_{0}\right)}^{2}$$

where $k_{a}$ is the angle spring constant, and $\theta_{0}$ is the equilibrium angle. Here, we fixed $k_{a}$ for cross-linked network at $250\;k_{B}T/{\mathrm{rad}}^{2}$, and we set $k_{a}$ for the nanorod at 250, 25 and $2.5\;k_{B}T/{\mathrm{rad}}^{2}$, to modulate their flexibility. The equilibrium angle $\theta_{0}$ is fixed at 180°. The positions of cross-linked joints of networks are restrained via a self-spring potential with a harmonic constant of $1\;k_{B}T/\sigma^{2}$, to prevent the collective movement of cross-linked network.

The motion of network and nanorod beads is controlled via Langevin equation:

(4) $$m_{i}{\ddot{r}}_{i}=-▿\sum_{j}U\left(r_{ij}\right)-m_{i}\zeta {\dot{r}}_{i}+F_{i}^{R}\left(t\right)$$

where $r_{i}$ represents the position vector, ζ is the friction coefficient, and we set it to 1.0. $F_{i}^{R}$ is the random force, which satisfies the fluctuation-dissipation theorem,

(5) $$\langle F_{i}^{R}\left(t\right)F_{j}^{R}\left(t^{\prime }\right)\rangle =6\zeta k_{B}T\delta_{ij}\delta \left(t-t^{\prime }\right)$$

the coupling of dissipation and random forces serves as an effective thermostat to control the system temperature and mimic the solvent effect implicitly. It should be noted that, the smooth energy landscape of coarse-grained model, as well as implicit solvent system would accelerate the system dynamics inevitably, resulting in faster dynamics than the real experimental systems. Nevertheless, the qualitative diffusion behavior and the comparison of dynamical properties of nanorods with different effective lengths and rigidities could be still analyzed.

The simulation box had a fixed volume, with dimensions of 54σ along the *y*-direction and 48σ along the *x*- and *z*-directions. Periodic boundary conditions (PBCs) were applied in all the three dimensions. In each simulation system, the total number of CG beads of thin nanorods was maintained at approximately 800, while the number of CG beads of thick nanorods was roughly 400, in order to guarantee the constant total volume fraction of nanorods. The number of nanorods was adjusted accordingly to account for the varying lengths and effective diameters. We carried out the simulations for $1\times 10^{8}$ steps, with a time step δt=0.005τ, where $\tau =\sigma \sqrt{m/\epsilon }$ is the time unit of the simulation system. All simulations were performed using the LAMMPS [37] software package (version: 29 Oct 2020). For each system, five independent parallel runs with different random seeds for the Langevin equation were conducted to obtain averaged results.

## 3. Results and Discussions

### 3.1. Translational Diffusion of Thin Nanorods

**Figure 2:**
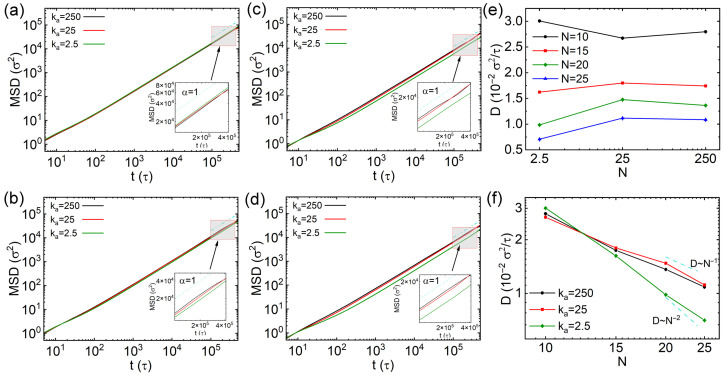
(**a**–**d**) Mean square displacements (MSDs) of thin nanorods with varying rigidities: (**a**) The results from nanorods with 10 CG beads, (**b**–**d**) the results from nanorods with 15, 20 and 25 beads, respectively. (**e**,**f**) Diffusion coefficients of the nanorods as functions of rigidities and different number of beads (*N*), respectively. Error bars are smaller than the symbol size.

First, we investigated the diffusion mechanisms of thin nanorods. The mean square displacements (MSDs) of the centers of mass of nanorods were calculated to quantify their diffusion behavior. The MSD is defined as

(6) $$\mathrm{MSD}=\langle {\left(r\left(t_{0}+t\right)-r\left(t_{0}\right)\right)}^{2}\rangle$$

where $r(t_{0}+t)$ denotes the position vector at the time $t_{0}+t$. The MSD results of thin nanorods with different number of CG beads (*N*) and rigidities are exhibited in Figure 2a–d, with insets displaying the data between $1\times 10^{5}$ and $4\times 10^{5}\tau$. Overall, the MSD values decrease with increasing *N*, which is attributed to greater steric hindrance experienced by the nanorods with longer effective length. For nanorods with 10 CG beads (N=10), the MSD is slightly larger when $k_{a}=2.5\;k_{B}T/{\mathrm{rad}}^{2}$ (inset of Figure 2a), indicating that softer thin nanorods diffuse faster than semiflexible and rigid ones ($k_{a}=25$ and $250\;k_{B}T/{\mathrm{rad}}^{2}$, respectively). However, if the nanorods contain more CG beads (N=15, 20 and 25), those with $k_{a}=2.5\;k_{B}T/{\mathrm{rad}}^{2}$ diffuse more slowly than their semiflexible and rigid counterparts, as shown in Figure 2b–d. To compare diffusion across different rigidities more quantitatively, we computed the diffusion coefficient *D* by linearly fitting the slope of the MSD curves over the time interval from $1\times 10^{5}\tau$ to $4\times 10^{5}\tau$. The diffusion coefficient is calculated via $D=\lim_{t\to \infty }\left(\mathrm{MSD}/2dt\right)$, where *d* indicates the dimension for calculating *D*. Thus, the equation changes to $D=\lim_{t\to \infty }\left(\mathrm{MSD}/6t\right)$ for calculating three-dimensional diffusion. The results of diffusion coefficients *D* are exhibited in Figure 2e,f. While the diffusivities of semiflexible and rigid nanorods are similar across all values of *N*, the dependence of *D* on rigidity exhibits diverse non-monotonic trends that varies with *N*: the semiflexible nanorods ($k_{a}=25\;k_{B}T/{\mathrm{rad}}^{2}$) diffuse most slowly for N=10; on the other hand, the semiflexible nanorods show fastest diffusion for N=15, 20 and 25. This behavior may be explained by the relationship between the effective length of the nanorods and the mesh size of the cross-linked network. A fully stretched nanorod with N=10 has a length of 10σ, which is still shorter than the overall space diagonal of the cell ($\sqrt{3}\times 6\sigma$), but the effective lengths of nanorods including more CG beads (N=15, 20 and 25) are longer than the overall space diagonal of the cell in cross-linked network. We further calculated the mean square end to end distances of nanorods with different *N* values and rigidities, and the results are exhibited in Figure S1a in Supplementary Materials. The relationship between the effective length of nanorods and the cell size might lead to varying diffusion tendencies of nanorods with their rigidities. Furthermore, *D* decreases approximately inversely with the number of beads *N* for rigid and semiflexible nanorods with $k_{a}=250\;k_{B}T$ and $25\;k_{B}T$. The scaling law is similar as the results of rigid nanorods in polymer melts [27]. In contrast, for soft nanorods with $k_{a}=2.5\;k_{B}T$, the reduction of *D* becomes significantly more pronounced with increasing *N*, following a scaling of $D∼N^{-1.5}$. This distinct scaling suggests that the diffusion of soft nanorods exhibits a crossover from Rouse-like to reptation-like dynamics, reminiscent of polymer chains [38]. This behavior is attributed to the local constraints imposed by cross-links.

**Figure 3:**
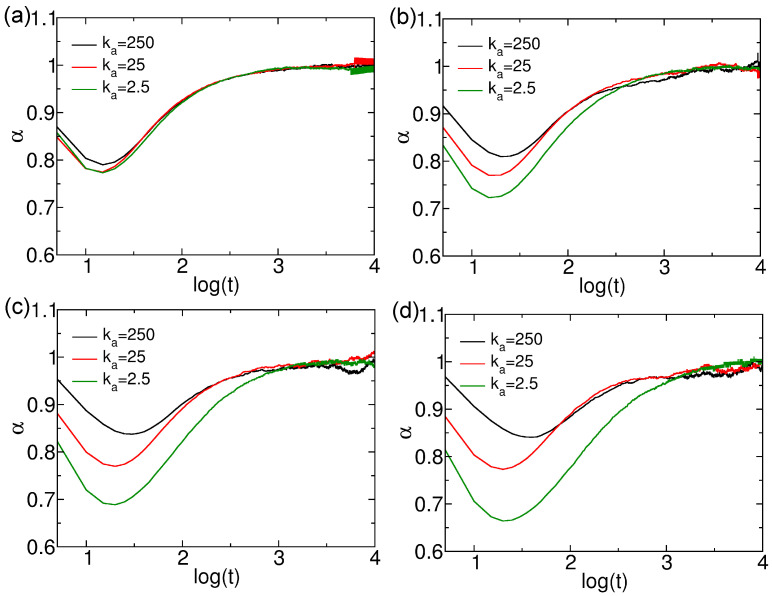
Anomalous diffusion exponent α of thin nanorods with different rigidities. Results are shown for nanorods composed of (**a**) 10, (**b**) 15, (**c**) 20, and (**d**) 25 CG beads, respectively.

If the microscopic nanoparticles are confined in the network architecture, the linear relationship between MSD and time might break down, leading to the anomalous diffusion described by $\mathrm{MSD}∼t^{\alpha }$, where α represents the anomalous diffusion coefficient. The diffusion falls into sub-diffusion regime if α<1, α>1 corresponds to the situation of super-diffusion usually induced by external perturbation (α=2 means ballistic diffusion), and α=1 indicates normal diffusion. The anomalous diffusion exponents α of thin nanorods with different number of CG beads and rigidities are presented in Figure 3. For all simulated nanorods, sub-diffusion is observed at short time scales (<$1\times 10^{3}\tau$), after which the diffusion transitions to a normal regime at longer times. The evolutions of α values of thin nanorods with N=10 are similar across different $k_{a}$ values of nanorods (Figure 3a). In contrast, nanorods with more CG beads (N=15, 20, and 25) exhibit more pronounced sub-diffusion as their rigidity decreases (Figure 3b–d). Notably, the time scales at which the minimum α values occur are similar for nanorods with different *N* and rigidity values. Furthermore, for rigid nanorods with $k_{a}=250\;k_{B}T/{\mathrm{rad}}^{2}$, the minimum α increases with *N*, whereas, for soft nanorods with $k_{a}=2.5\;k_{B}T/{\mathrm{rad}}^{2}$, the minimum α decreases as *N* increases. For example, α decays to roughly 0.65 in soft nanorods with N=25; this value is similar to the short time polymer chain dynamics before relaxation [38,39]. This indicates an opposing dependence of the sub-diffusion effect on the number of CG beads for nanorods of identical rigidity. Thus, the confinement effect on the nanorods depends on both their rigidities and *N* values. We also investigated the diffusion properties of innermost beads and end beads of thin nanorods for identifying the Rouse and reptation dynamics of nanorods, especially the soft ones [38,39]. The results are available in Figures S2 and S3 in Supplementary Materials, with the corresponding discussions.

**Figure 4:**
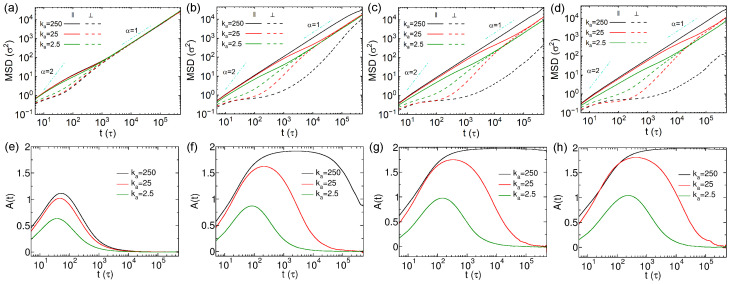
(**a**–**d**) Mean square displacements (MSDs) parallel and perpendicular to the major axes of thin nanorods with different rigidities. Results are shown for nanorods composed of (**a**) 10, (**b**) 15, (**c**) 20, and (**d**) 25 CG beads, respectively. (**e**–**h**) Anisotropic diffusion parameter A(t) for the nanorods with 10, 15, 20 and 25 beads, respectively.

The non-monotonic dependence of the diffusion coefficient on the rigidity of thin nanorods, along with the contrasting sub-diffusion behavior observed in rigid versus soft thin nanorods, suggests a potential trade-off among multiple factors influencing nanorod diffusion in cross-linked networks. Thus, we decomposed the MSDs of nanorods into two components: one parallel and the other perpendicular to the major axes. The calculation procedure of the unit vector of major axis of nanorod is detailed in Supplementary Materials. The different components of MSDs are calculated by

(7) $$\begin{array}{cc} {\mathrm{MSD}}_{\|} & =\langle {\left(\left(r\left(t_{0}+t\right)-r\left(t_{0}\right)\right)\cdot e_{\|}\left(t_{0}\right)\right)}^{2}\rangle \\ {\mathrm{MSD}}_{⊥1} & =\langle {\left(\left(r\left(t_{0}+t\right)-r\left(t_{0}\right)\right)\cdot e_{⊥1}\left(t_{0}\right)\right)}^{2}\rangle \\ {\mathrm{MSD}}_{⊥2} & =\langle {\left(\left(r\left(t_{0}+t\right)-r\left(t_{0}\right)\right)\cdot e_{⊥2}\left(t_{0}\right)\right)}^{2}\rangle \end{array}$$

where $e_{\|}$ is the unit vector parallel to the major axis of nanorod, $e_{⊥1}$ and $e_{⊥2}$ are the unit vectors perpendicular to it. The perpendicular MSD was taken as the average of ${\mathrm{MSD}}_{⊥1}$ and ${\mathrm{MSD}}_{⊥2}$. The MSDs parallel and perpendicular to the major axes of thin nanorods are shown in Figure 4a–d. The parallel MSDs slightly exceed the perpendicular ones for nanorods with N=10 at short times, with the two components converging around $1\times 10^{3}\tau$ (Figure 4a). In order to quantify the anisotropic diffusion of nanorods more effectively, we calculated the anisotropic diffusion parameter, which is defined as follows [24]:

(8) $$A\left(t\right)=\frac{3\langle {\mathrm{MSD}}_{\|}\left(t\right)\rangle }{\langle \mathrm{MSD}\left(t\right)\rangle }-1$$

where A(t)=0 corresponds to isotropic diffusion, and the deviation of A(t) from 0 indicates anisotropic diffusion. Specifically, A(t)=2 represents that the nanorods only diffuse along their major axes. For the nanorods with N=10, A(t) values derived from MSDs shown in Figure 2a and Figure 4a are plotted in Figure 4e. The peak in A(t) decreases as the nanorod softens, indicating that softer rods diffuse more isotropically compared to semiflexible and rigid ones. For the nanorods with N=15, the decoupling between parallel and perpendicular MSDs becomes more pronounced (Figure 4b). The two MSD components of rigid nanorods with $k_{a}=250\;k_{B}T/{\mathrm{rad}}^{2}$ remain separated throughout the simulations, indicating persistent anisotropic diffusion within the simulation time scale. Analogous to the N=10 case, the parallel MSDs of nanorods with N=15 also exhibit super-diffusion effect following initial ballistic diffusion. In contrast, the perpendicular MSDs display more noticeable sub-diffusion between $10^{1}$ and $10^{3}\tau$, especially for the rigid nanorods with $k_{a}=250\;k_{B}T/{\mathrm{rad}}^{2}$. The corresponding A(t) results further confirm strong diffusion anisotropy in rigid nanorods with N=15, with values close to 2 between $10^{3}$ and $10^{4}\tau$ (Figure 4f). The anisotropic diffusion effect decreases with rigidity of nanorods with N=15, and they vanish at long time scales for the semiflexible and soft nanorods ($k_{a}=25$ and $2.5\;k_{B}T/{\mathrm{rad}}^{2}$). The pronounced sub-diffusion perpendicular to the major axis and the strong anisotropy in rigid nanorods are attributed to steric hindrance from the cross-linked network. Since the effective length of the rigid nanorod with N=15 exceeds the mesh size of the network (Figure S1a), diffusion perpendicular to the major axes of nanorods is strongly suppressed. The rigid nanorods with N=20 and 25 exhibit more evident anisotropic diffusion effect, with the strong sub-diffusion behavior of these rigid nanorods leading to very small perpendicular MSDs (Figure 4c,d). Their A(t) values remain near 2 from $10^{3}\tau$ to the end of the simulations (Figure 4g,h). The A(t) results drop as the nanorods with N=20 and 25 become softer, due to the reduced parallel MSDs, which is because of larger cross-sectional areas (see $\lambda_{2}$ and $\lambda_{3}$ in Table S1), as well as the increase of perpendicular MSDs, indicating that the anisotropic diffusion effect weakens monotonically as the decrease of rigidity of nanorods. This opposite trend in the two MSD components with varying rigidity leads to the non-monotonic variation in diffusivity *D* with $k_{a}$ (Figure 2c,d). We also computed the anomalous diffusion exponents (α) for both parallel and perpendicular directions based on the MSDs in Figure 4a–d, and the results are shown in Figure S4. The observed short-time anisotropic diffusion and long-time isotropic diffusion are consistent with previous findings by Han and co-workers [40]. The representative trajectory videos for the diffusion mechanisms of thin nanorods containing 10 and 25 beads with different rigidities are available in Supplementary Materials.

**Figure 5:**
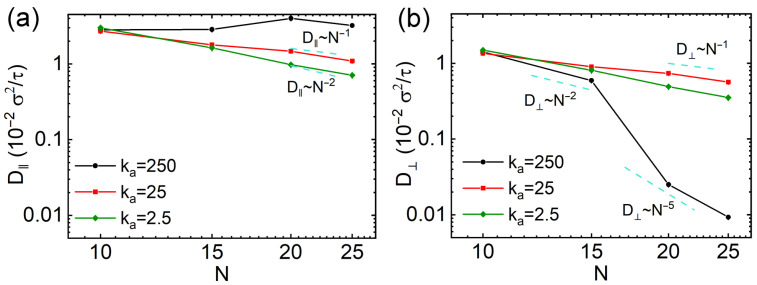
Diffusion coefficients parallels to (**a**) and perpendicular to (**b**) the major axes of thin nanorods with different rigidities as a function of *N* values.

We also calculated the diffusion coefficients along the directions parallel and perpendicular to the major axes of nanorods. For one-dimensional motion along the axis, the parallel diffusion coefficient is defined as $D_{\|}=\lim_{t\to \infty }\left({\mathrm{MSD}}_{\|}/2t\right)$. For two-dimensional diffusion on the cross-sectional plane perpendicular to the axis, the perpendicular coefficient is $D_{⊥}=\lim_{t\to \infty }\left({\mathrm{MSD}}_{⊥}/4t\right)$. The $D_{\|}$ and $D_{⊥}$ were also obtained by fitting the slopes of ${\mathrm{MSD}}_{\|}$ and ${\mathrm{MSD}}_{⊥}$ over the interval $1\times 10^{5}$ to $4\times 10^{5}\tau$, respectively, to represent the anisotropic dynamics of nanorods at long time scales. The results of $D_{\|}$ and $D_{⊥}$ of nanorods with different rigidities and *N* values are exhibited in Figure 5a,b, respectively. The $D_{\|}$ and $D_{⊥}$ values of semiflexible and soft nanorods are similar, consistent with their long-time isotropic diffusion behavior (Figure 4). Their scaling laws also align with those derived from the total MSD results (Figure 2). However, rigid nanorods exhibit markedly different trends: $D_{\|}$ of rigid nanorods depends non-monotonically on their *N* values, resembling earlier observations on translational diffusion of rod-like particles in obstacles [41,42]. On the other hand, $D_{⊥}$ decreases with increasing *N*, approximately following $D_{⊥}∼N^{-2}$ for N≤15, then $D_{⊥}$ reduces extremely rapidly for longer rigid nanorods with scaling roughly as $D_{⊥}∼N^{-5}$. This shift in scaling is attributed to the increased effective length of nanorods with N≥15, which exceeds the overall space diagonal of the cell, resulting in very strong constraint on the diffusion perpendicular to the major axes of nanorods. These findings are consistent with prior studies on nanorod diffusion in polymer melts [27,43].

**Figure 6:**
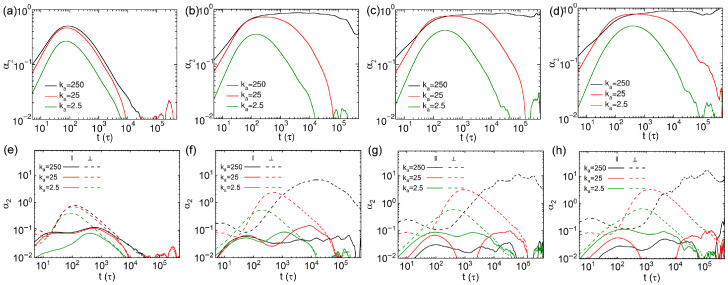
Non-Gaussian parameters $\alpha_{2}$ for thin nanorods with different *N* and rigidites. Results of three dimensional $\alpha_{2}$ values for nanorods composed of (**a**) 10, (**b**) 15, (**c**) 20, and (**d**) 25 CG beads, respectively; (**e**–**h**) $\alpha_{2}$ values parallel and perpendicular to the major axes of nanorods, corresponding to the systems shown in (**a**–**d**).

The restraint imposed by the cross-linked network on the nanorods leads to anomalous diffusion, with particularly pronounced sub-diffusion behavior observed in the direction perpendicular to the major axes of nanorods. This suggests that the diffusion of nanorods within the cross-linked network deviates from the Brownian regime. To characterize the dynamical heterogeneity of the nanorods, we calculated the non-Gaussian parameter $\alpha_{2}$, which is defined as follows [44,45]:

(9) $$\alpha_{2}\left(t\right)=\frac{\langle r^{4}\left(t\right)\rangle }{\left(1+2/d\right){\langle r^{2}\left(t\right)\rangle }^{2}}-1$$

where *d* denotes the dimensionality considered in the calculation of $\alpha_{2}$. A deviation of $\alpha_{2}\left(t\right)$ from zero indicates that the distribution of displacements does not follow Gaussian distribution, which corresponds to heterogeneous dynamics, while a decay to zero corresponds to Gaussian behavior, indicating normal diffusion. The results of three-dimensional non-Gaussian parameters ($\alpha_{2}$) of thin nanorods with different *N* and $k_{a}$ values are displayed in Figure 6a–d. The $\alpha_{2}$ values reach maxima around 80τ for the nanorods with N=10 (Figure 6a), and the times for the $\alpha_{2}$ values to reach maxima show little dependence on the rigidities of nanorods with N=10. The overall $\alpha_{2}$ results are smaller with decreasing rigidity of nanorods, indicating that softer nanorods exhibit diffusion behavior closest to the Brownian regime. For longer nanorods (N≥15), whose effective lengths exceed the spatial diagonal of a network mesh cell, the peak $\alpha_{2}$ values for rigid ($k_{a}=250$) and semiflexible ($k_{a}=25\;k_{B}T/{\mathrm{rad}}^{2}$) nanorods are similar (Figure 6b–d). Moreover, the $\alpha_{2}$ values of the rigid nanorods do not decay to zero, implying that the Brownian regime does not occur within our simulation time scale for the rigid nanorods with longer effective lengths. In contrast, the $\alpha_{2}$ values for the semiflexible and soft nanorods vanish to 0 at long time scales, with a faster decay observed for soft nanorods, confirming the onset of Brownian diffusion. Notably, the time scales at which $\alpha_{2}$ values reach maxima coincide with those observed for the corresponding anisotropic diffusion parameter A(t) results (Figure 4e–h). This consistency suggests that anisotropic diffusion and heterogeneous dynamics share common underlying influencing factors.

In analogy to the analysis of MSD, we also decompose the non-Gaussian parameter of nanorod into the components of parallel and perpendicular to the major axis of nanorod. For the calculation of $\alpha_{2}$ parallel to the major axis, the parameter *d* in Equation (9) was set to 1, corresponding to the one-dimensional diffusion along this direction. On the other hand, *d* was set to 2 for the calculation of $\alpha_{2}$ for the perpendicular component, as diffusion occurs in the two-dimensional plane normal to the major axis. The results of $\alpha_{2}$ of thin nanorods in the parallel and perpendicular directions are presented in Figure 6e–h. The perpendicular $\alpha_{2}$ results are significantly larger than those in parallel direction, for all the nanorods we simulated. The parallel $\alpha_{2}$ values are smaller than those of the semiflexible and rigid ones for soft nanorods with N=10 (Figure 6e). However, the tendency starts to shift for the nanorods with N=15, in which the $\alpha_{2}$ results become comparable across all the three rigidities (Figure 6f). For longer nanorods with N=20 and 25, the parallel $\alpha_{2}$ values of the soft nanorods exceed those of the more rigid ones (Figure 6g,h), attributed to larger cross-sectional areas for the soft nanorods (Table S1) hindering the translational diffusion along their major axes. The $\alpha_{2}$ results in the perpendicular direction decrease monotonically as the nanorods become softer, and those from the rigid nanorods do not decay to zero in the systems including nanorods with N≥15. These results suggest that the observed anisotropic diffusion and heterogeneous dynamics arise mainly from the confinement imposed by the cross-linked networks, which promote diffusion along the major axes of nanorods, particularly in the case of rigid nanorods.

### 3.2. Rotational Diffusion of Thin Nanorods

**Figure 7:**
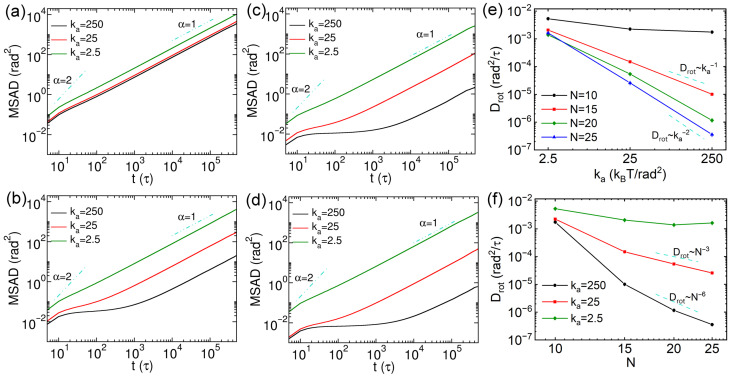
(**a**–**d**) Mean square angular displacements (MSADs) of thin nanorods with varying rigidities, with N=10, 15, 20 and 25, respectively. (**e**,**f**) Rotational diffusion coefficients of the nanorods as functions of rigidities and *N* values, respectively. The error bars are smaller than the symbol size.

The anisotropic shape of nanorods results in the more pronounced influence of confinement from the cross-linked network on their rotational dynamics. To quantify this effect, we compute the mean square angular displacements (MSADs) to characterize the rotational diffusion of the nanorods. The MSAD is defined as follows [29,46,47,48,49]:

(10) $$\mathrm{MSAD}=\langle {\left(\vec{\varphi }\left(t_{0}+t\right)-\vec{\varphi }\left(t_{0}\right)\right)}^{2}\rangle$$

where $\vec{\varphi }\left(t\right)$ is the total angular displacement, given by $\vec{\varphi }\left(t\right)=\int_{0}^{t}\Delta \vec{\varphi }\left(t^{\prime }\right)dt^{\prime }$, the magnitude of $\Delta \vec{\varphi }\left(t^{\prime }\right)$ is obtained from $\cos^{-1}\left(e_{\|}\left(t+\delta t\right)\cdot e_{\|}\left(t\right)\right)$, and the cross product $e_{\|}\left(t+\delta t\right)\times e_{\|}\left(t\right)$ corresponds to the instantaneous axis of rotation. The rotational diffusion coefficient $D_{rot}$ was extracted by linear fitting with MSAD/4t over the time interval $1\times 10^{5}$ to $4\times 10^{5}\tau$, in which MSADs exhibit a linear dependence on simulation time. The results of MSADs of thin nanorods with different rigidities and *N* values are exhibited in Figure 7a–d, and the corresponding $D_{rot}$ results are presented in Figure 7e,f. The rotational dynamics slow down as the increase of rigidity of nanorods at a fixed *N*. The MSAD curves exhibit ballistic rotational diffusion at short time scales (<10τ). Then, the rotations of nanorods with N=10 turn to normal diffusion at longer time scales. A similar normal diffusion regime after ballistic motion is observed for soft nanorods with N=15, however, semiflexible and rigid nanorods with N=15 display sub-diffusive rotational behavior, which becomes more pronounced in rigid systems (Figure 7b). This effect is further reflected in the anomalous rotational diffusion exponent results (obtained via $\mathrm{MSAD}∼t^{\alpha_{rot}}$) shown in Figure S5b. A comparable trend is observed for nanorods with N=20 and 25, and rigid nanorods again exhibit more evident sub-diffusion of rotation (Figure 7c,d), as also indicated by the $\alpha_{rot}$ results in Figure S5c,d. The rotational diffusion coefficients $D_{rot}$ decrease with increasing rigidities of nanorods, and $D_{rot}$ values become smaller for rigid and semiflexible nanorods with longer effective lengths (Figure 7e). However, the $D_{rot}$ values do not vary systematically with *N* values of soft nanorods. In addition, the relationship between $D_{rot}$ and $k_{a}$ for N=10 nanorods differs from that for longer rods. For N=15, 20, and 25, a clear scaling law of $D_{rot}∼k_{a}^{l}$ is observed, with the exponent *l* ranging between −1 and −2 and decreasing as the nanorod length increases. The $D_{rot}$ values of nanorods with N=10 show no clear dependence on their rigidities, likely because their effective lengths are smaller than the space diagonal of network cell. In contrast, steric hindrance has a more significant effect on the rotational dynamics of longer nanorods (N=15, 20, and 25). The $D_{rot}$ values as a function of *N* values of nanorods are presented in Figure 7f, which indicates that $D_{rot}$ reduces more rapidly with increasing *N* for more rigid nanorods. Moreover, both semiflexible and rigid nanorods with N≥15 exhibit scaling behavior, following $D_{rot}∼N^{-3}$ and $D_{rot}∼N^{-6}$, respectively. These scaling exponents are larger than those reported for nanorod rotation in polymer blends [27,43], which may be attributed to the strong confining effect imposed by the stable cross-links of the network.

The sub-diffusion rotational dynamics also indicate the heterogeneous rotations of nanorods in cross-linked networks. Similar to the analysis of translational motion, we evaluated the non-Gaussian parameter for rotational diffusion of nanorods ($\alpha_{2\_rot}$), defined as

(11) $$\alpha_{2\_rot}\left(t\right)=\frac{3\langle \Delta \varphi^{4}\left(t\right)\rangle }{5{\langle \Delta \varphi^{2}\left(t\right)\rangle }^{2}}-1$$

**Figure 8:**
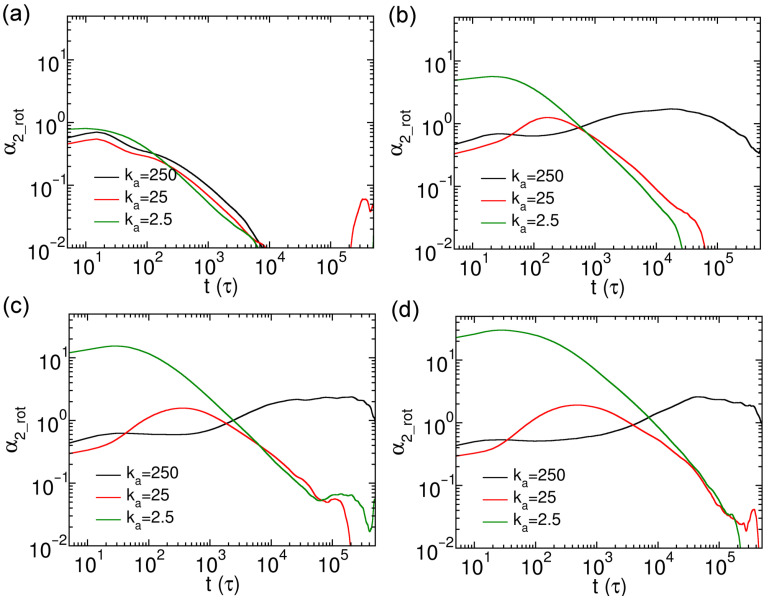
Rotational non-Gaussian parameters $\alpha_{2\_rot}$ for thin nanorods with different *N* and rigidities. Results are shown for nanorods composed of (**a**) 10, (**b**) 15, (**c**) 20, and (**d**) 25 CG beads, respectively.

The non-Gaussian parameter for rotational diffusion of thin nanorods ($\alpha_{2\_rot}$) are exhibited in Figure 8, which indicates the heterogeneous rotational dynamics of nanorods. The $\alpha_{2\_rot}$ results of the nanorods with N=10 show no clear dependence on their rigidities; all the curves in Figure 8a suggest only weakly heterogeneous rotational dynamics, attributed to the relatively shorter length than the space diagonal of the cell. In contrast, the rotational heterogeneity becomes more pronounced for the nanorods with longer effective lengths (N=15, 20 and 25). First, the time scales at which $\alpha_{2\_rot}$ reach maxima increase with *N* for the nanorods with same rigidity. On the other hand, the characteristic time scales also lengthen with increasing rigidities at the same *N* value (Figure 8b–d). In addition, the maximum values of $\alpha_{2\_rot}$ of soft nanorods are significantly larger than those of semiflexible and rigid ones (Figure 8b–d). This may be due to the larger cross-sectional area of soft nanorods (Table S1), which increases their probability of being constrained by the cross-linked network at short time scales, thereby enhancing the effect of heterogeneous rotation.

### 3.3. Diffusion of Thick Nanorods

**Figure 9:**
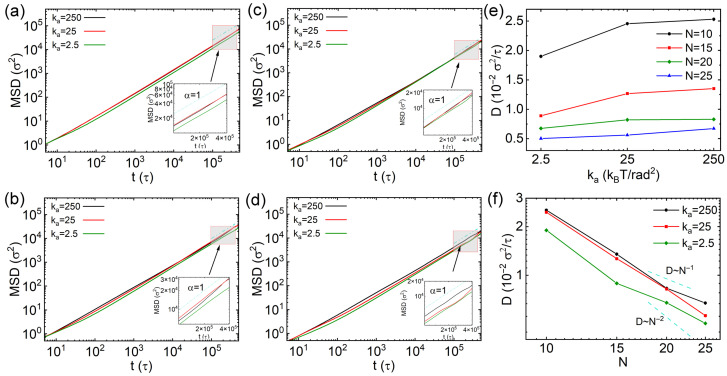
(**a**–**d**) Mean square displacements (MSDs) of thick nanorods with varying rigidities: (**a**) The results from nanorods with 10 CG beads, and (**b**–**d**) the results from nanorods with 15, 20 and 25 beads, respectively. (**e**,**f**) Diffusion coefficients of the nanorods as functions of rigidities and different number of beads (*N*), respectively. Error bars are smaller than the symbol size.

We investigated the diffusion mechanisms of thick nanorods, which have an effective cross-sectional area twice that of thin nanorods, by calculating their mean squared displacements (MSDs) and diffusion coefficients in cross-linked networks. The results of MSDs of thick nanorods with different rigidities and *N* values are presented in Figure 9a–d. Unlike the thin nanorods, comparisons of MSDs for thick nanorods with N=10 reveal distinct behavior: soft thick nanorods with N=10 diffuse the slowest among the three rigidities (Figure 9a), a trend also reflected in their diffusion coefficients (Figure 9e,f). This contrasts with the behavior of thin nanorods, for which the soft ones diffuse fastest (Figure 2a). Moreover, soft thick nanorods with N=10 exhibit more pronounced sub-diffusion, as indicated by their lower anomalous diffusion exponent α results (Figure S6a). Their slower dynamics can be attributed to the larger effective cross-sectional area, which raises the energy barrier for their diffusion through network cells and enhances local motion perpendicular to their major axes. For thick nanorods with N≥15, MSDs also decrease with increasing flexibility, with soft thick nanorods again displaying markedly slow dynamics (Figure 9b–d). The anomalous diffusion behaviors of these longer thick nanorods are similar to those of thin nanorods, except that sub-diffusion emerges at earlier simulation times (Figure S6b–d). Diffusion coefficients of thick nanorods follow scaling laws with *N* values, falling between $D∼N^{-1}$ and $D∼N^{-2}$ (Figure 9f). Notably, all three rigidity types exhibit similar scaling trends, in contrast to the distinct behaviors observed for thin nanorods (Figure 2f).

**Figure 10:**
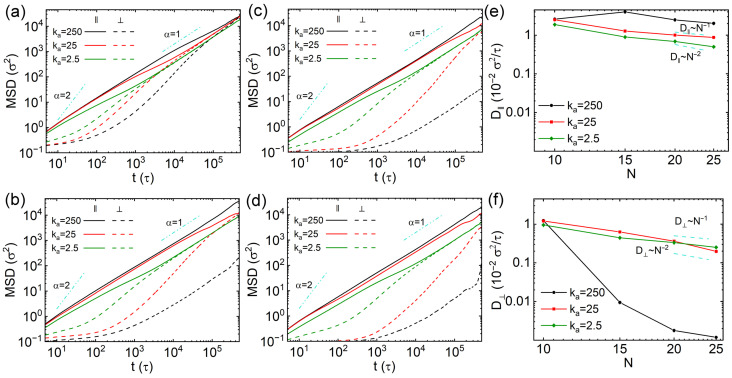
(**a**–**d**) Mean square displacements (MSD) parallel and perpendicular to the major axes of the thick nanorods with different rigidities. Results are shown for nanorods composed of (**a**) 10, (**b**) 15, (**c**) 20, and (**d**) 25 CG beads, respectively. (**e**,**f**) Diffusion coefficients of the nanorods as functions of rigidities and different number of beads (*N*), respectively. Error bars are smaller than the symbol size.

Similar to thin nanorods, we investigated the anisotropic diffusion of thick nanorods by computing the mean-square displacements (MSDs) parallel and perpendicular to their major axes. For thick nanorods with N=10, diffusion anisotropy is markedly more pronounced than in thin nanorods, as reflected by considerably smaller perpendicular MSDs (Figure 4a and Figure 10a). A strong sub-diffusive behavior is also observed in the perpendicular direction at short time scales, which is attributed to the larger effective cross-sectional area of the thick nanorods. This trend is further supported by the anisotropic parameter A(t) results of thick nanorods with N=10, which are shown in Figure S7a. Rigid nanorods with N=15 exhibit pronounced diffusion anisotropy, with their parallel and perpendicular MSDs displaying parallel trends in double-logarithmic plots (Figure 10b), resulting in A(t) approaching 2 and remaining nearly constant throughout the simulation (Figure S7b). Semiflexible and soft nanorods with N=15 also show clear anisotropic diffusion at shorter time scales, but their dynamics become isotropic at longer time scales (Figure 10b). Notably, the crossover time from anisotropic to isotropic diffusion occurs later for thick nanorods than for thin ones. For thick nanorods with N=20 and 25, anisotropic diffusion effects remain more evident compared to thin nanorods of the same rigidity and *N* value (Figure 10c,d and Figure S7c,d). The representative trajectory videos for the diffusion of thick nanorods with N=10 and 25 are available in Supplementary Materials. The dynamical heterogeneity of thick nanorods with different *N* values and rigidities are quantified by the non-Gaussian parameter $\alpha_{2}$, and the results of three-dimensional $\alpha_{2}$ results, along with their parallel and perpendicular components, are shown in Figure S8. The results indicate that the thick nanorods with large effective cross-sectional areas exhibit more heterogeneous dynamics than thin nanorods, in both parallel and perpendicular directions. We also calculated the eigenvalues of mean square radius gyration tensors of thick nanorods, and the results are shown in Table S2. The larger cross-sectional area leads to relatively smaller eigenvalues perpendicular to the major axis compared to thin nanorods, especially for semiflexible and soft configurations. This reduction is due to stronger confinement imposed by the cross-linked network in the direction perpendicular to the major axes of nanorods.

The parallel and perpendicular diffusion coefficients ($D_{\|}$ and $D_{⊥}$) for thick nanorods are presented in Figure 10e,f, obtained by fitting the linear regimes of the corresponding MSDs. Similar to thin nanorods, the $D_{\|}$ values for rigid thick nanorods show no clear dependence on the number of beads *N* (Figure 5a and Figure 10e). For semiflexible and soft thick nanorods, $D_{\|}$ follows scaling laws ranging between $D∼N^{-1}$ and $D∼N^{-2}$, with similar trends observed for both the two rigidities. The $D_{⊥}$ for semiflexible thick nanorods with N=10 and 15 exceeds that of soft nanorods (Figure 10f), consistent with the behavior of *D* and $D_{\|}$. The comparison of $D_{⊥}$ of semiflexible and soft nanorods starts to switch at N=20, and it shows a reverse relationship with $D_{⊥}$ of semiflexible nanorods smaller than that of soft nanorods with N=25. The scaling of $D_{⊥}$ for both semiflexible and soft thick nanorods also lies between $D∼N^{-1}$ and $D∼N^{-2}$. In contrast, $D_{⊥}$ for rigid thick nanorods decreases rapidly with increasing *N*, and it remains lower than the values observed for rigid thin nanorods (Figure 5).

**Figure 11:**
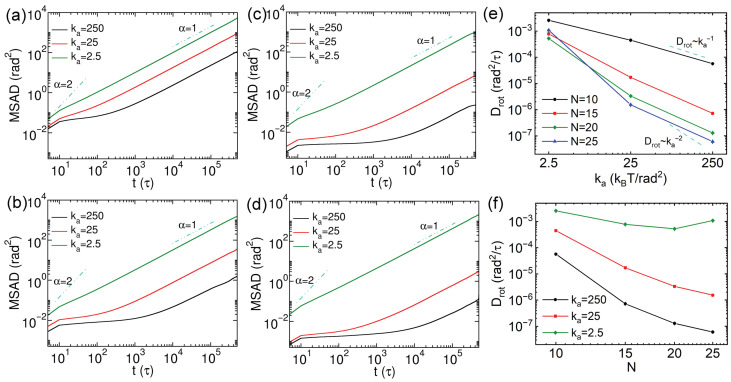
(**a**–**d**) Mean square angular displacements (MSADs) of thick nanorods with varying rigidities, with N=10, 15, 20 and 25, respectively. (**e**,**f**) Rotational diffusion coefficients of the nanorods as functions of rigidities and *N* values, respectively. The error bars are smaller than the symbol size.

The rotational dynamics of thick nanorods were also characterized via calculating MSADs, and their rotational diffusion coefficients ($D_{rot}$) were extracted from the linear portion of the MSAD curves via slope fitting. Figure 11 presents the MSAD results for thick nanorods with varying *N* and rigidities, along with the corresponding $D_{rot}$ values. For rigid thick nanorods with N=10, a distinct sub-diffusive regime is observed from $10^{1}$ to $10^{3}\tau$ (Figure 11a), which is more pronounced than that of thin nanorods with the same *N* and rigidity. Differences in MSADs among the three stiffness levels are also more evident for thick nanorods, as reflected in $D_{rot}$ of nanorods with N=10 (Figure 11e,f), yielding a scaling relation of $D∼k_{a}^{-1}$. For thick nanorods with N≥15, the MSAD trends resemble those of thin nanorods, though with slower rotational dynamics. The $D_{rot}$ values for these systems follow scaling laws between $D∼k_{a}^{-1}$ and $D∼k_{a}^{-2}$, except that the soft nanorods with N=25, which rotate faster than those with N=15 and 20 (Figure 11e). This deviation may arise from their greater effective length and more complex conformational fluctuations. The $D_{rot}$ values decrease monotonically with increasing *N* for the semiflexible and rigid thick nanorods; however, no clear scaling behavior is found for the dependence of $D_{rot}$ on *N*. This may result from the strongly constrained rotational dynamics imposed by the cross-linked network. The rotational non-Gaussian parameters for thick nanorods are shown in Figure S9. They indicate that rotational heterogeneity is more pronounced in rigid and semiflexible thick nanorods compared to thin ones. For soft thick versus thin nanorods, the comparison is less clear because rotation is influenced by both ends of the nanorod as well as by intricate changes in intramolecular configuration.

## 4. Conclusions and Outlooks

In this study, we employed CGMD simulations to explore the translational and rotational diffusion behaviors and mechanisms of nanorods in cross-linked networks. Our results reveal a weak non-monotonic dependence of diffusivity of thin nanorods on their rigidities, which arises from a trade-off between the opposing dependencies of parallel and perpendicular MSDs on the rigidities of nanorods. The thick nanorods exhibit slower dynamics with their decreased rigidities. The diffusions of both the thin and thick nanorods obey scaling laws with the beads contained in nanorods. The anisotropic diffusion behavior of nanorods with moving along their long axis preferentially becomes more pronounced with increasing length and rigidity of nanorods, especially for thick nanorods. The parallel and perpendicular diffusion coefficients of semiflexible and soft nanorods depend on *N* values with scaling laws. The translational diffusion behaviors of nanorods exhibit dynamical heterogeneity, mainly attributed to the confinement imposed by the cross-linked network, which particularly restricts motion perpendicular to the major axes of nanorods. The rotational dynamics are slower in longer and more rigid nanorods, with the latter also displaying pronounced sub-diffusion rotation behavior. The effect of sub-diffusive rotation becomes more pronounced in thick nanorods. Furthermore, the rotational diffusion coefficient follows a scaling-law decay with increasing rigidities for thin nanorods whose effective lengths exceed the mesh size of the network, and this phenomenon exists in all the thick nanorods we investigated. The nanorods exhibit heterogeneous rotational diffusions, including the longer time scale heterogeneity of rotation for more rigid nanorods, as well as more pronounced heterogeneity in the systems with softer nanorods. We anticipate that these findings will inspire further investigation into nanoparticle–polymer network composites, aiding the design of functional materials and providing insight into related biological systems.

In real polymer network systems, the architecture is typically non-uniform and non-cubic; the specific pattern and topology of the network also influence the diffusion of nanoparticles. Several studies have examined the role of network functionality, for example, diamond network with tetra-functionality [14,50], on the diffusion of isotropic nanoparticles. Dai and co-workers found that the functionality, genus and degree of cross-linked networks exhibit a heterogeneous free energy barrier for particle hopping among different cages [15]. Therefore, it is anticipated that the dynamics of rod-shaped nanoparticles in complex cross-linked networks, which exhibit polydispersity in functionality, pore size, and other structural features, will be diverse and merit future investigation.

## Data Availability

The original contributions presented in this study are included in the article/Supplementary Material. Further inquiries can be directed to the corresponding author.

## Supplementary Materials

The following are available online at https://www.mdpi.com/article/10.3390/polym18010003/s1. The MSDs of innermost and end beads of nanorods, the method for determining the major axis of nanorod are available in Supplementary Materials. The additional figures and tables are listed as follows: Figure S1: Mean square end to end distances ($\langle R_{ee}^{2}\rangle$) of thin nanorods (a) and thick nanorods (b) with varying *N* and rigidities. Figure S2: (a–d) Mean square displacements of innermost beads of thin nanorods with different rigidities, (a) is the results of nanorods with N=10, (b), (c) and (d) exhibit the results of nanorods with N=15, 20 and 25, respectively. (e–h) Anomalous diffusion exponents corresponding to the MSDs shown in (a–d). Figure S3: Mean square displacements of end beads of soft nanorods with various *N* values; (b) the ratios between the MSDs of end beads and innermost beads of soft nanorods. Figure S4: Anomalous diffusion exponents (α) parallel and perpendicular to the major axes of thin nanorods with different rigidities. (a–d) are the results of nanorods with 10, 15, 20 and 25 CG beads, respectively. Figure S5: Anomalous rotational diffusion exponents ($\alpha_{rot}$) of thin nanorods with different rigidities. (a–d) are the results of nanorods with 10, 15, 20 and 25 CG beads, respectively. Figure S6: Anomalous rotational diffusion exponents (α) of thick nanorods with different rigidities. (a–d) are the results of nanorods with 10, 15, 20 and 25 CG beads, respectively. Figure S7: Anisotropic diffusion parameters A(t) of thick nanorods with different rigidities. (a–d) are the results of nanorods with 10, 15, 20 and 25 CG beads, respectively. Figure S8: Non-Gaussian parameters $\alpha_{2}$ for thick nanorods with different *N* and rigidites. (a–d) Results of three dimensional $\alpha_{2}$ values for nanorods composed of (a) 10, (b) 15, (c) 20, and (d) 25 CG beads, respectively; (e–h) $\alpha_{2}$ values parallel and perpendicular to the major axes of nanorods. Figure S9: Rotational non-Gaussian parameters $\alpha_{2\_rot}$ for thick nanorods with different *N* and rigidities. Results are shown for nanorods composed of (a) 10, (b) 15, (c) 20, and (d) 25 CG beads, respectively. Table S1: Eigenvalues of mean square radius of gyration tensors of thin nanorods, the two eigenvalues $\lambda_{2}$ and $\lambda_{3}$ linked to the unit vectors perpendicular to the major axes can reflect the cross-sectional areas of nanorods qualitatively. Table S2: Eigenvalues of mean square radius of gyration tensors of thick nanorods. The supporting video article is available in Supplementary Materials. References [38,39,51,52,53] are cited in the Supplementary Materials.

## Abbreviations

The following abbreviations are used in this manuscript:

PEG	polyethylene glycol
CGMD	coarse-grained molecular dynamics
LJ	Lennard-Jones
PBC	periodic boundary condition
MSD	mean square displacement
MSAD	mean square angular displacement
