# Supplementary material for: Diffusion of Nanorods with Various Lengths and Rigidities in Cross-Linked Networks
Source: Polymers (Basel). 2025 Dec 19;18(1):3. doi: 10.3390/polym18010003 (PMC12787724; doi:10.3390/polym18010003)
Supplement: Supplementary file 1 [file polymers-18-00003-s001.zip › SI.pdf]

Supplementary Material: Diffusion of Nanorods with Various Lengths and Rigidities in Cross-Linked Networks

Bin Li [1,*] and Pingcuozhuoga [1,2]

1 School of Chemical Engineering and Technology, Sun Yat-sen University, Zhuhai 519082, China

2 State Grid Xizang Electric Power Research Institute, Lhasa 850000, China

Correspondence: libin76@mail.sysu.edu.cn

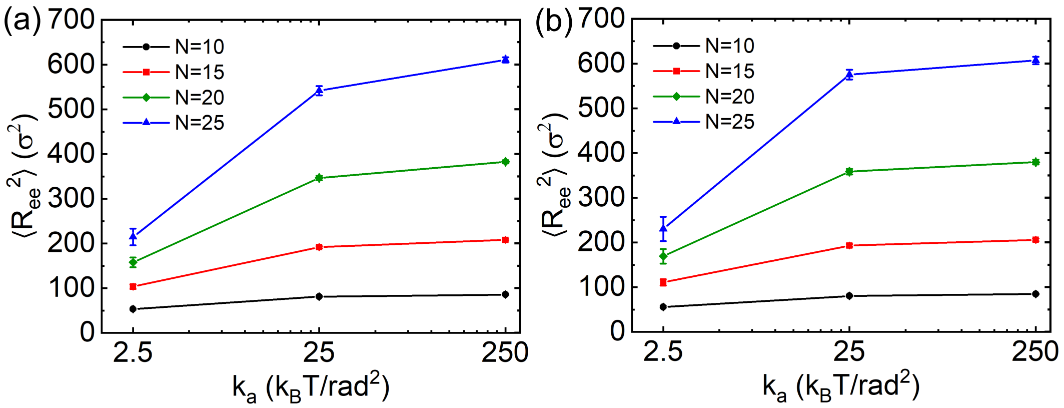

Figure S1: Mean square end to end distances ($\langle R_{ee}^2 \rangle$) of thin nanorods (a) and thick nanorods (b) with varying N and rigidities.

Diffusion of innermost and end coarse-grained beads of thin nanorods

We calculated the MSD of innermost CG beads of thin nanorods ($\mathrm{MSD}_{N/2}$), for characterizing the restraint of cross-linked network on the different parts of nanorods. The position of innermost CG beads of nanorods with 10 and 20 beads is defined as the center of the two innermost beads. The $\mathrm{MSD}_{N/2}$ results of nanorods with different N values and rigidities are exhibited in Figure S2a-d, which are almost same as those of centers of mass of nanorods at long time scales. However, the $\mathrm{MSD}_{N/2}$ results at short time scales ($0 \sim 100\tau$) are slightly larger than those of centers of mass of nanorods, which results in smaller anomalous diffusion coefficient results $\alpha_{N/2}$ (Figure S2e-h), especially for soft nanorods with $k_a = 2.5k_\mathrm{B}T/\mathrm{rad}^2$. For example, the minimum of $\alpha_{N/2}$ of soft nanorods with $k_a = 2.5k_\mathrm{B}T/\mathrm{rad}^2$ is roughly 0.45, which also indicates that the diffusion of longer soft nanorods changes from Rouse-like to reptation-like dynamics of polymer chains.[1]

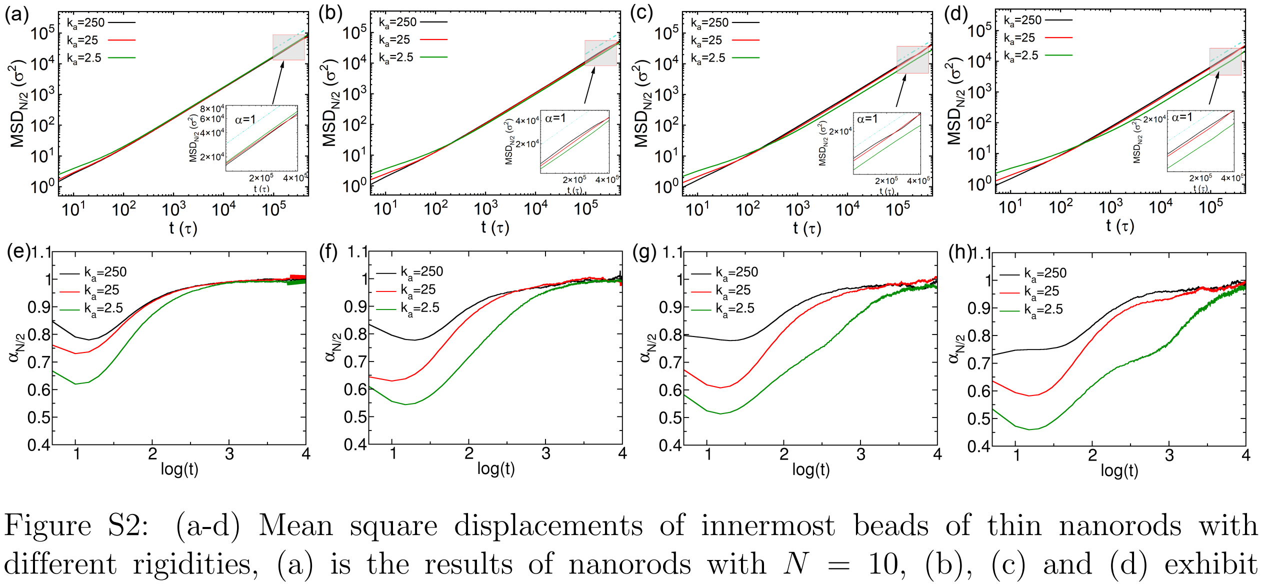

Figure S2: (a-d) Mean square displacements of innermost beads of thin nanorods with different rigidities, (a) is the results of nanorods with $N = 10$, (b), (c) and (d) exhibit the results of nanorods with $N = 15$, 20 and 25, respectively. (e-h) Anomalous diffusion exponents corresponding to the MSDs shown in (a-d).

We also calculated the MSD of end beads of thin nanorods (MSD_e), and the results of MSD_e of soft nanorods with different N values are shown in Figure S3a. The MSD_e for rigid and semiflexible nanorods are not shown here due to the similar trend as $MSD_{N/2}$. In order to characterize the local dynamical mechanism of soft thin nanorods, we calculate the ratio of $MSD_e/MSD_{N/2}$, and the results are exhibited in Figure S3b. The diffusion of end beads of soft nanorods is faster than that of innermost beads at short time scales, especially for the nanorods with $N = 25$, and all the ratios decay to 1 at long time scales, indicating identical dynamics of different parts of nanorods. The maxima of $MSD_e/MSD_{N/2}$ results increase as the N values of soft nanorods, and $MSD_e/MSD_{N/2}$ reaches about three for the soft nanorods with $N = 25$. The maximum of $MSD_e/MSD_{N/2}$ is expected equal to two for the Rouse dynamics of polymer chain, the results of longer soft nanorods with $N = 20$ and 25 are beyond the limit of Rouse regime (Figure S3b), which indicates the potential reptation-like dynamics.[1,2]

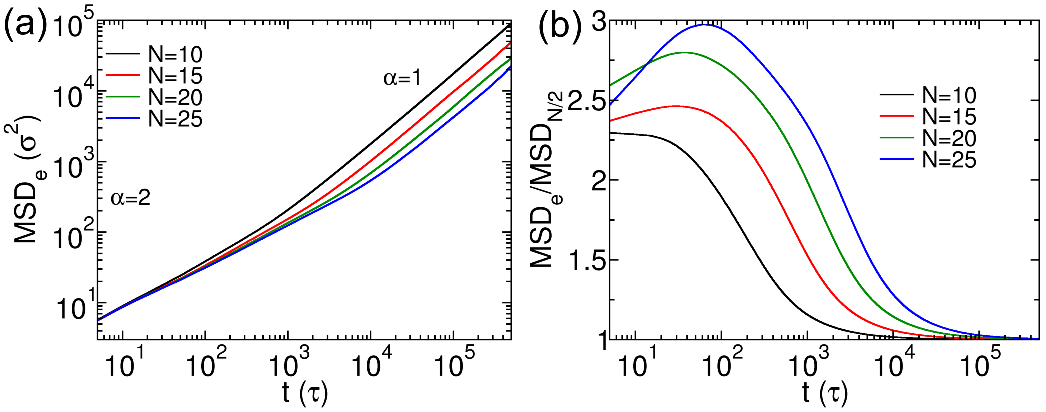

Figure S3: Mean square displacements of end beads of soft nanorods with various N values;(b) the ratios between the MSDs of end beads and innermost beads of soft nanorods.

The approach for calculating the major axis of nanorod

The unit vectors parallel and perpendicular to the major axis of the nanorod were evaluated via the Jacobian transformation of its radius of gyration tensor, which is described as,[2–5]

$$A = \begin{bmatrix} S_{xx} & S_{xy} & S_{xz} \\ S_{yx} & S_{yy} & S_{yz} \\ S_{zx} & S_{zy} & S_{zz} \end{bmatrix} \rightarrow \begin{bmatrix} \lambda_1 \\ \lambda_2 \\ \lambda_3 \end{bmatrix} \begin{bmatrix} S_1 & S_4 & S_7 \\ S_2 & S_5 & S_8 \\ S_3 & S_6 & S_9 \end{bmatrix} \tag{1}$$

with $S_{ab} = \frac{1}{n}\sum_{i=1}^{n}(a_i - a_{cm})(a_i - b_{cm})$, where the indices a and b denote x, y, or z components of the coordinates of CG beads, a_{cm} and b_{cm} are the centers of mass of nanorods at x, y or z direction. The tensor is then diagonalized to find its eigenvalues λ_1, λ_2, and λ_3. These eigenvalues are sorted such that $\lambda_1 > \lambda_2 \approx \lambda_3$, consistent with the expected geometry of a nanorod. Hence, the eigenvector linked to λ_1 is identified as the major axis $\mathbf{e}_{\parallel}$ of nanorod, and the remaining two eigenvectors as the perpendicular axes $\mathbf{e}_{\perp 1}$ and $\mathbf{e}_{\perp 2}$.

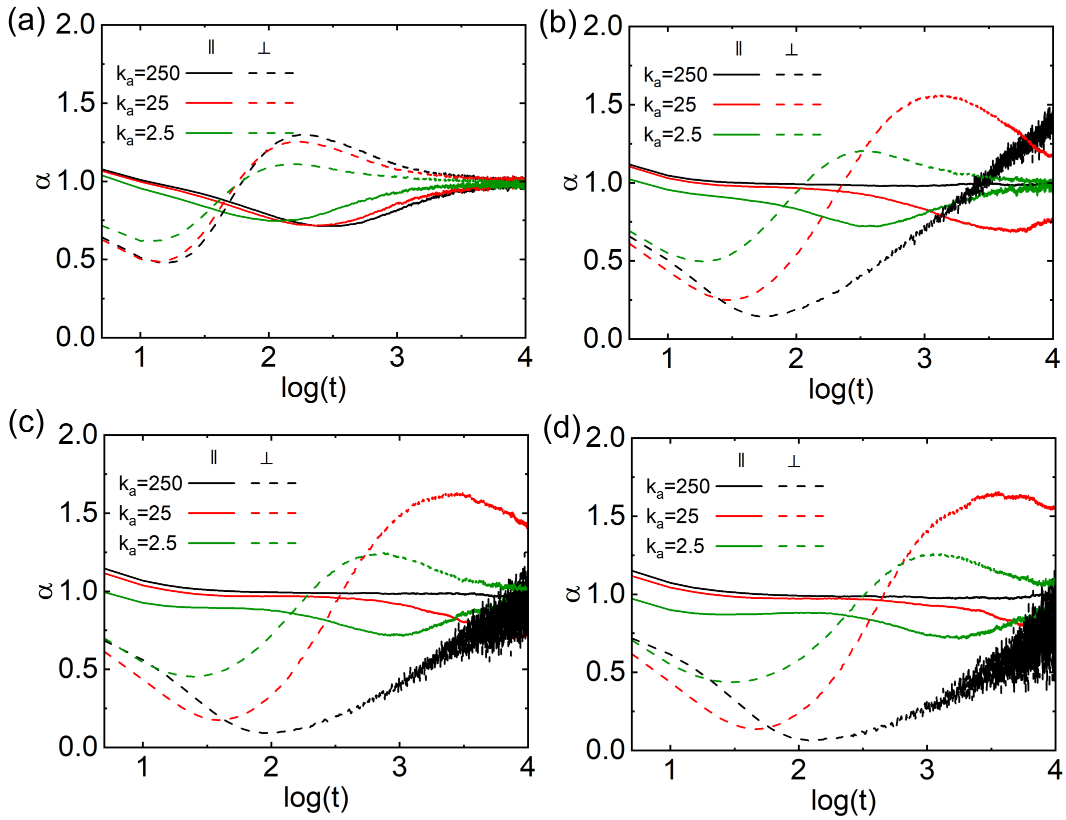

Figure S4: Anomalous diffusion exponents (α) parallel and perpendicular to the major axes of thin nanorods with different rigidities. (a-d) are the results of nanorods with 10, 15, 20 and 25 CG beads, respectively.

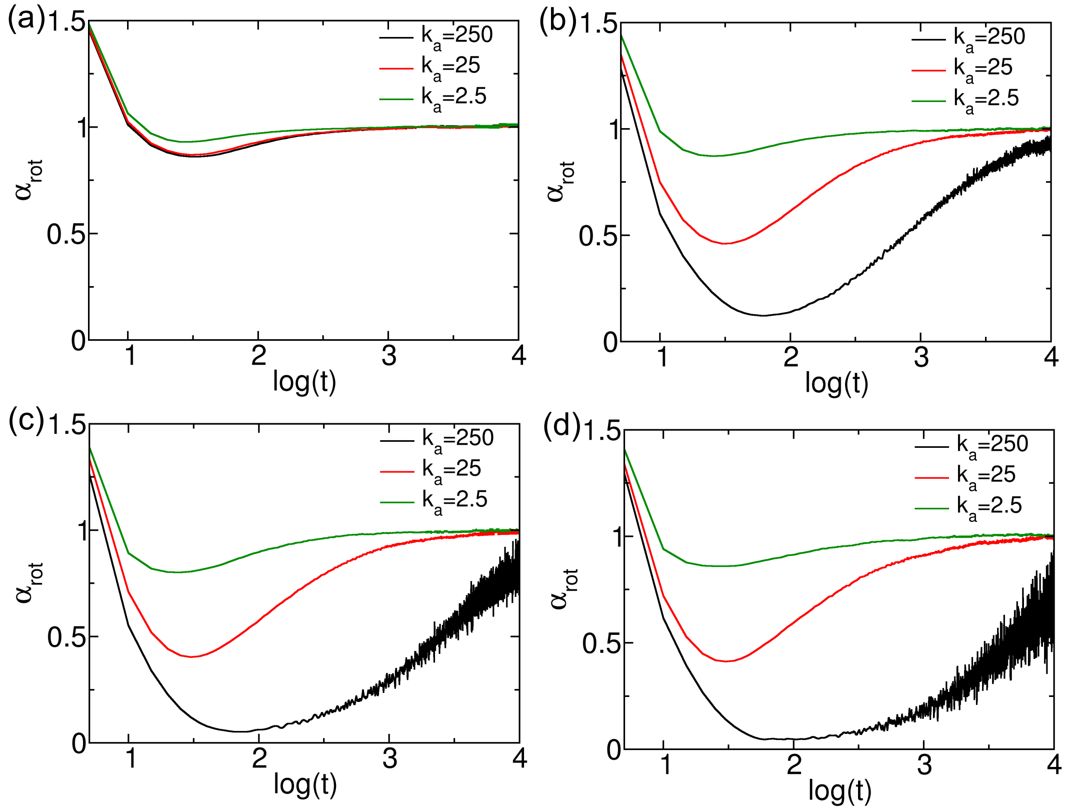

Figure S5: Anomalous rotational diffusion exponents (α_{rot}) of thin nanorods with different rigidities. (a-d) are the results of nanorods with 10, 15, 20 and 25 CG beads, respectively.

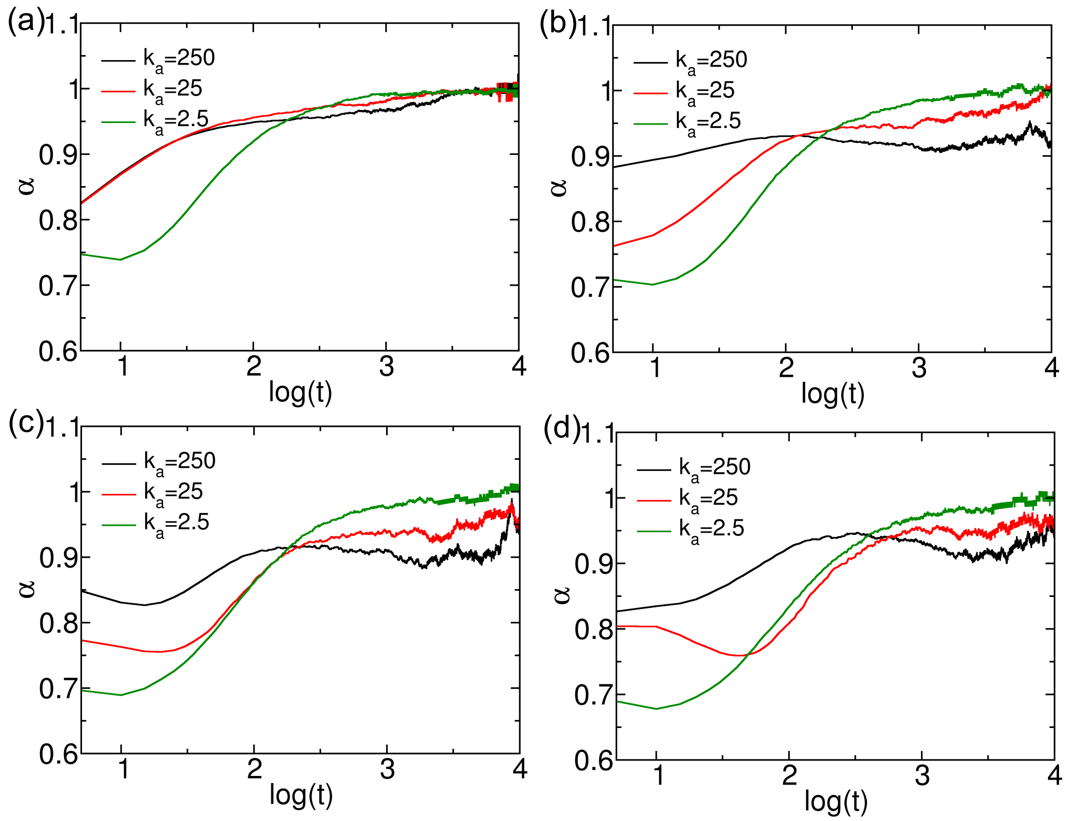

Figure S6: Anomalous rotational diffusion exponents (α) of thick nanorods with different rigidities. (a-d) are the results of nanorods with 10, 15, 20 and 25 CG beads, respectively.

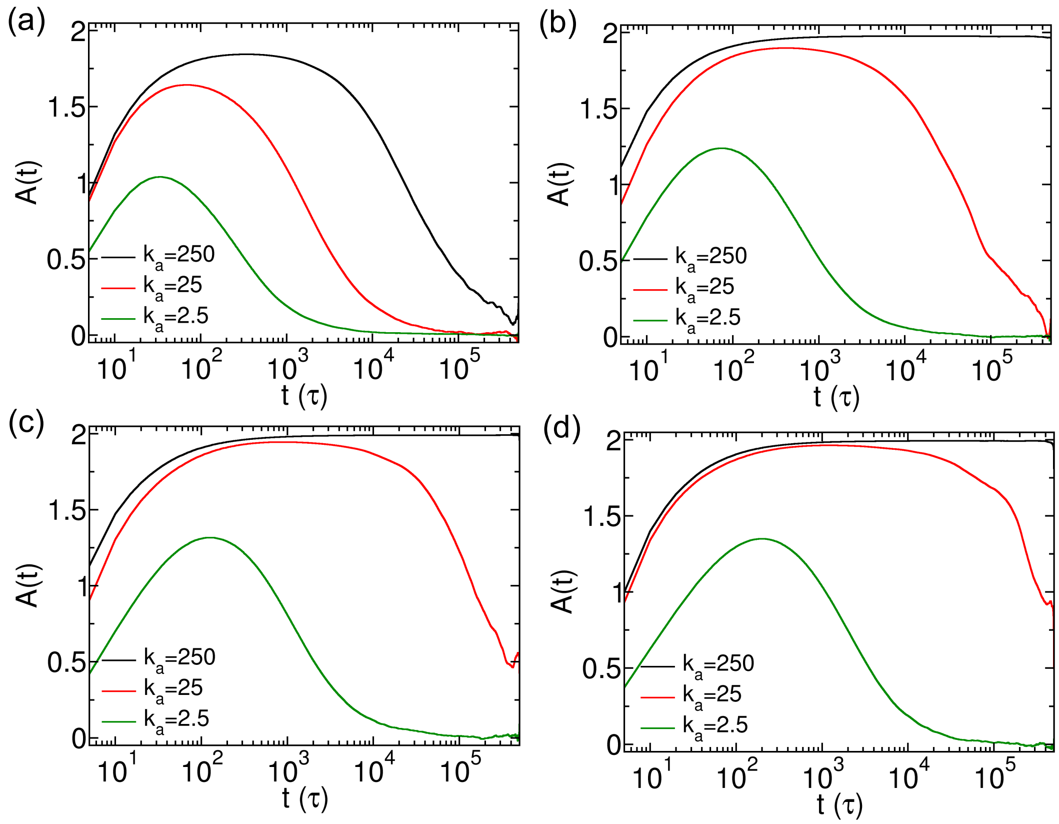

Figure S7: Anisotropic diffusion parameters $A(t)$ of thick nanorods with different rigidities.(a-d) are the results of nanorods with 10, 15, 20 and 25 CG beads, respectively.

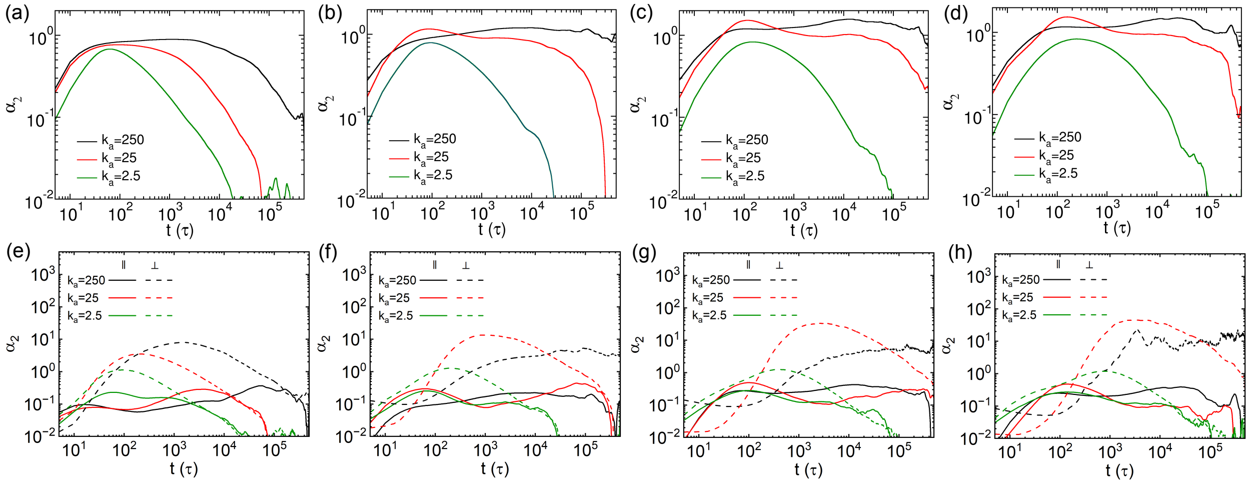

Figure S8: Non-Gaussian parameters α_2 for thick nanorods with different N and rigidites.(a-d) Results of three dimensional α_2 values for nanorods composed of (a) 10, (b) 15, (c) 20, and (d) 25 CG beads, respectively; (e-h) α_2 values parallel and perpendicular to the major axes of nanorods.

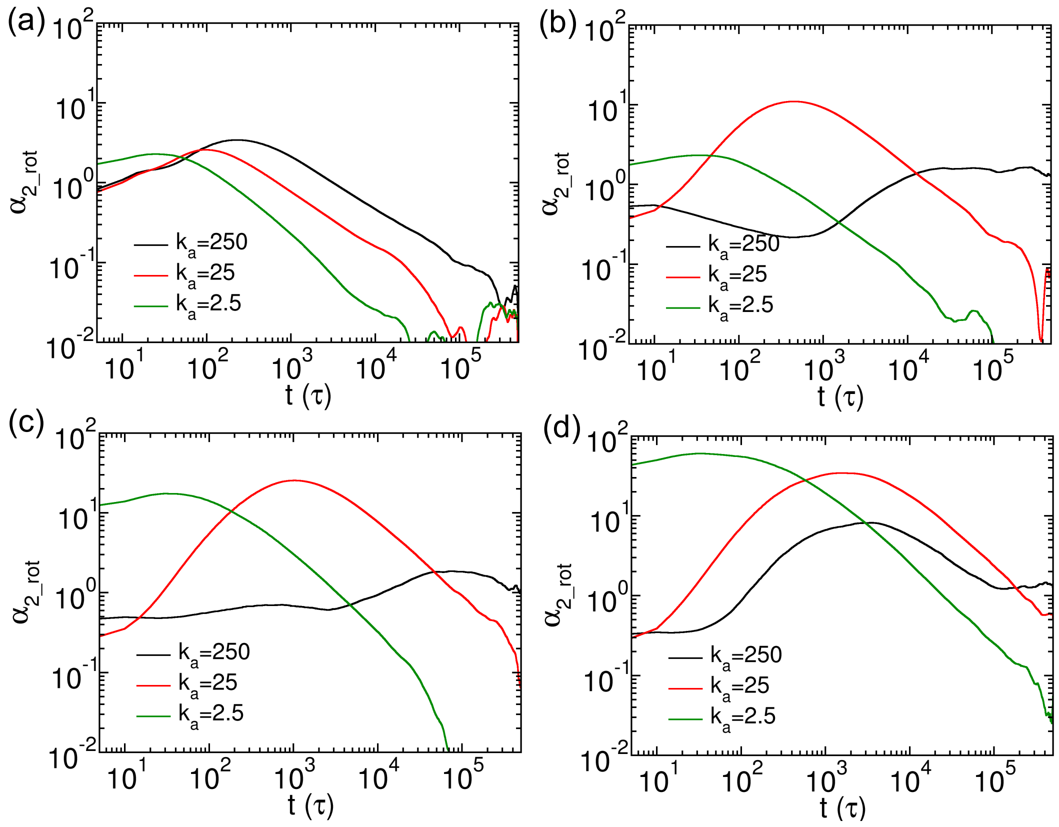

Figure S9: Rotational non-Gaussian parameters $\alpha_{2\ rot}$ for thick nanorods with different N and rigidities. Results are shown for nanorods composed of (a) 10, (b) 15, (c) 20, and (d) 25 CG beads, respectively.

Table S1: Eigenvalues of mean square radius of gyration tensors of thin nanorods, the two eigenvalues λ_2 and λ_3 linked to the unit vectors perpendicular to the major axes can reflect the cross-sectional areas of nanorods qualitatively.

(a) λ_1, λ_2 and λ_3 for the nanorods with $N = 10$ and different rigidities.

$k_a(k_\mathrm{B}T/rad^2)$	250	25	2.5
$\lambda_1(\sigma^2)$	8.76	8.37	5.90
$\lambda_2(\sigma^2)$	9.39×10^{-3}	8.93×10^{-2}	0.469
$\lambda_3(\sigma^2)$	7.71×10^{-4}	9.39×10^{-3}	6.01×10^{-2}

(b) λ_1, λ_2 and λ_3 for the nanorods with $N = 15$ and different rigidities.

$k_a(k_\mathrm{B}T/rad^2)$	250	25	2.5
$\lambda_1(\sigma^2)$	19.9	18.6	11.2
$\lambda_2(\sigma^2)$	2.91×10^{-2}	0.263	1.21
$\lambda_3(\sigma^2)$	2.44×10^{-3}	2.31×10^{-2}	0.166

(c) λ_1, λ_2 and λ_3 for the nanorods with $N = 20$ and different rigidities.

$k_a(k_\mathrm{B}T/rad^2)$	250	25	2.5
$\lambda_1(\sigma^2)$	35.4	32.6	17.2
$\lambda_2(\sigma^2)$	5.96×10^{-2}	0.534	2.20
$\lambda_3(\sigma^2)$	5.35×10^{-3}	4.76×10^{-2}	0.338

(d) λ_1, λ_2 and λ_3 for the nanorods with $N = 25$ and different rigidities.

$k_a(k_\mathrm{B}T/rad^2)$	250	25	2.5
$\lambda_1(\sigma^2)$	55.3	50.2	23.6
$\lambda_2(\sigma^2)$	9.16×10^{-2}	0.957	3.35
$\lambda_3(\sigma^2)$	9.50×10^{-3}	8.15×10^{-2}	0.575

Table S2: Eigenvalues of mean square radius of gyration tensors of thick nanorods.

(a) λ_1, λ_2 and λ_3 for the nanorods with $N = 10$ and different rigidities.

$k_a(k_\mathrm{B}T/rad^2)$	250	25	2.5
$\lambda_1(\sigma^2)$	8.68	8.28	6.12
$\lambda_2(\sigma^2)$	9.26×10^{-3}	8.51×10^{-2}	0.394
$\lambda_3(\sigma^2)$	7.65×10^{-4}	7.42×10^{-3}	5.38×10^{-2}

(b) λ_1, λ_2 and λ_3 for the nanorods with $N = 15$ and different rigidities.

$k_a(k_\mathrm{B}T/rad^2)$	250	25	2.5
$\lambda_1(\sigma^2)$	19.7	18.6	11.7
$\lambda_2(\sigma^2)$	2.64×10^{-2}	0.185	1.18
$\lambda_3(\sigma^2)$	2.74×10^{-3}	1.99×10^{-2}	0.106

(c) λ_1, λ_2 and λ_3 for the nanorods with $N = 20$ and different rigidities.

$k_a(k_\mathrm{B}T/rad^2)$	250	25	2.5
$\lambda_1(\sigma^2)$	35.1	33.5	18.0
$\lambda_2(\sigma^2)$	5.43×10^{-2}	0.224	2.22
$\lambda_3(\sigma^2)$	5.39×10^{-3}	3.54×10^{-2}	0.286

(d) λ_1, λ_2 and λ_3 for the nanorods with $N = 25$ and different rigidities.

$k_a(k_\mathrm{B}T/rad^2)$	250	25	2.5
$\lambda_1(\sigma^2)$	55.0	52.6	24.9
$\lambda_2(\sigma^2)$	6.34×10^{-2}	0.267	3.37
$\lambda_3(\sigma^2)$	9.20×10^{-3}	4.61×10^{-2}	0.517

References

(1) Golmohammadi, N.; Boland-Hemmat, M.; Barahmand, S.; Eslami, H. Coarse-grained molecular dynamics simulations of poly(ethylene terephthalate). *J. Chem. Phys.* **2020**, *152*, 114901.

(2) Kreer, T.; Baschnagel, J.; Müller, M.; Binder, K. Monte Carlo Simulation of Long Chain Polymer Melts: Crossover from Rouse to Reptation Dynamics. *Macromolecules* **2001**, *34*, 1105–1117.

(3) Li, B.; Zhao, L.; Qian, H.-J.; Lu, Z.-Y. Coarse-grained simulation study on the self-assembly of miktoarm star-like block copolymers in various solvent conditions. *Soft Matter* **2014**, *10*, 2245–2252.

(4) Lin, C.-M.; Chen, Y.-Z.; Sheng, Y.-J.; Tsao, H.-K. Effects of macromolecular architecture on the micellization behavior of complex block copolymers. *React. Funct. Polym.* **2009**, *69*, 539–545.

(5) Zhang, J.-J.; Li, B. Self-Assembly of Star-Polyelectrolytes in Various Solution Conditions. *Macromolecules* **2024**, *57*, 396–408.
